# Demonstrating a new approach to planning and monitoring rural medical training distribution to meet population need in North West Queensland

**DOI:** 10.1186/s12913-018-3788-0

**Published:** 2018-12-22

**Authors:** Matthew R. McGrail, Deborah J. Russell, Belinda G. O’Sullivan, Carole Reeve, Lee Gasser, David Campbell

**Affiliations:** 1 University of Queensland, Rural Clinical School, 78 on Canning Street, Rockhampton, QLD 4700 Australia; 2 Northern Territory Medical Program, Flinders University, PO Box 41326, Casuarina, NT 0815 Australia; 3 Monash Rural Health, Monash University, 26 Mercy Street, Bendigo, VIC 3550 Australia; 4 College of Medicine and Dentistry, James Cook University, 1 James Cook Drive, Townsville, QLD 4811 Australia; 5 Australian College of Rural and Remote Medicine, GPO Box 2507, Brisbane, QLD 4001 Australia

**Keywords:** Workforce planning, Access, Rural health, Remote communities, GP training, Primary care, Health care equity, Decision making

## Abstract

**Background:**

Improving the health of rural populations requires developing a medical workforce with the right skills and a willingness to work in rural areas. A novel strategy for achieving this aim is to align medical training distribution with community need. This research describes an approach for planning and monitoring the distribution of general practice (GP) training posts to meet health needs across a dispersed geographic catchment.

**Methods:**

An assessment of the location of GP registrars in a large catchment of rural North West Queensland (across 11 sub-regions) in 2017 was made using national workforce supply, rurality and other indicators. These included (1): Index of Access –spatial accessibility (2); 10-year District of Workforce Shortage (DWS) (3); MMM (Modified Monash Model) rurality (4); SEIFA (Socio-Economic Indicator For Areas) (5); Indigenous population and (6) Population size. Distribution was determined relative to GP workforce supply measures and population health needs in each health sub-region of the catchment. An expert panel verified the approach and reliability of findings and discussed the results to inform planning.

**Results:**

378 registrars and 582 supervisors were well-distributed in two sub-regions; in contrast the distribution was below expected levels in three others. Almost a quarter of registrars (24%) were located in the poorest access areas (Index of Access) compared with 15% of the population located in these areas. Relative to the population size, registrars were proportionally over-represented in the most rural towns, those consistently rated as DWS or those with the poorest SEIFA value and highest Indigenous proportion.

**Conclusions:**

Current regional distribution was good, but individual town-level data further enabled the training provider to discuss the nuance of where and why more registrars (or supervisors) may be needed. The approach described enables distributed workforce planning and monitoring applicable in a range of contexts, with increased sensitivity for registrar distribution planning where most needed, supporting useful discussions about the potential causes and solutions. This evidence-based approach also enables training organisations to engage with local communities, health services and government to address the sustainable development of the long-term GP workforce in these towns.

## Background

Geographical maldistribution of clinicians remains a primary concern of health workforce planning worldwide [1]. A key objective of the Australian government is to develop a general practice workforce that aligns strongly with population health needs, especially in dispersed rural and remote areas with limited access to other specialists [2, 3]. Despite their poorer health outcomes, rural Australians continue to experience poorer access to primary care [4, 5].

In addressing this, since 2001 the Australian General Practice Training (AGPT) program has a requirement that at least 50% of general practice training occurs in rural areas (rural being defined as Australian Standard Geographical Classification – Remoteness Areas 2–5). This strategy aims to develop doctors with the right skills for rural primary care work, improve the distribution of general practitioners (GPs) both short-term and long-term and increase access to GP services in rural and remote areas [6]. Providing positive educational experiences in rural settings and clearer training pathways in rural practice are known to be effective strategies for influencing early-career GPs to choose a rural career [7, 8]. Training is mostly managed through one of nine Regional Training Organisations (RTOs). The RTOs deliver training in pre-defined catchments, with endpoints of fellowship of at least one of the Australian College of Rural and Remote Medicine or the Royal Australian College of General Practitioners. Each RTO has at least some rural catchment, which demands that they make decisions about how to best distribute rural training places [9]. However, to date few RTOs have applied workforce planning to the distribution of training to address the long-term workforce and population needs of their communities. Anecdotally, convenience of placements and perceived quality of teaching are prioritised and thus rural training tends to occur in the largest eligible rural population centres.

This project focused on a single RTO, Generalist Medical Training (GMT), which is part of James Cook University (JCU). GMT is the only RTO with an explicit vision to meet workforce needs by encouraging registrars to work in underserved areas. It is a distributed training organisation, having node offices in all of its sub-regions to better engage locally and gain an understanding of local needs. Whilst GMT is affiliated with JCU’s undergraduate medical program, it trains doctors from all university programs. JCU aims to develop a socially accountable workforce to meet the health care needs of North West Queensland [10], with 60% of JCU graduates working in rural areas in their first 7 years since graduation [11]. Generalist Medical Training (GMT) coordinates the placement of doctors training in general practice in North West Queensland. Their training footprint comprises around 94% of the geographic area of Queensland (1.63 million km^2^, which is more than twice the size of France). It includes most of Queensland’s regional, rural and remote communities and a population of over 1.5 million people (around 34% of Queensland’s population), including some 100,000 Aboriginal and Torres Strait Islander peoples. The population distribution in GMT’s catchment is 84% rural and 16% metropolitan, the latter being limited to one sub-region, the Sunshine Coast.

Training doctors in smaller rural communities has at least three key benefits: (1) there is an increased likelihood they will develop clinical skills that are most relevant to rural practice; (2) there is an increased likelihood they will be better prepared for living in smaller rural communities and (3) registrars comprise a large additional workforce during their training period to provide essential primary care services to populations who most need these services [7, 12, 13]. These benefits can translate into improved uptake of rural practice, especially in the region where training took place, improved retention and improved population health outcomes [14, 15]. However, the extent to which the current distribution of training posts for doctors training in general practice (henceforth ‘registrars’) aligns with workforce shortages and population need, has not previously been systematically explored.

New planning methods are needed for exploring this, using information about the distribution of training posts relative to local geography, medical workforce supply and population need (community characteristics) has the potential, when coupled with local expertise, to support targeted planning for distribution around community need [16, 17]. Understanding the value of such methods may also inform distributed workforce planning more broadly. This project aims to apply a range of indicators as an approach for planning rural GP training distribution (supervisors and registrars) relative to workforce access and population need for primary care services across a large geographic catchment.

## Methods

This project is a collaboration between independent rural health workforce researchers and JCU. This research primarily utilises administrative data, with two key datasets used: (1) listing of all GP registrars in 2017 (practice-level, identified town); (2) listing of all active GP supervisors in 2017 (practice-level, identified town). Additionally, matched data between the primary supervisor and the registrar were used. Registrars currently doing procedural training in hospitals were excluded as these doctors were not providing GP services and were required to work in a limited range of larger rural town hospitals during this training period.

The geographical footprint comprises 11 sub-regions that approximately align with the Queensland Hospital and Health Services regions. Table 1 contains a summary of the relative size, population and population density in each region. Assessment of the distribution of registrars and supervisors across this catchment was calculated at both the sub-regional and town levels, though the latter is anonymised in this paper.

**Table 1:** Characteristics of North West Queensland’s region

Sub-region	Area (‘000 km^2^)	Population^a^ (‘000)	Density (per km^2^)	Main towns
Cairns^b^	8	185	23.1	Cairns, Innisfail
Central Qld	114	206	1.8	Rockhampton, Gladstone, Yeppoon
Central West	396	12	0.03	Longreach
Mackay	90	167	1.9	Mackay, Bowen, Airlie Beach
North West	198	29	0.15	Mt Isa, Cloncurry
South West	319	26	0.08	Roma, Charleville
Sunshine Coast	10	353	35.3	Sunshine Coast, Gympie, Nambour
Tablelands^b^	135	47	0.35	Mareeba, Atherton
Torres & Cape	130	23	0.18	Weipa, Thursday Island
Townsville	189	222	1.2	Townsville, Charters Towers, Ayr
Wide Bay	37	201	5.4	Bundaberg, Hervey Bay, Maryborough

^a^Population counts as per the 2011 Australian census

^b^Cairns and Hinterland sub-region (see Fig. 1) is separated into two sub-regions by the regional training organisation (Cairns, Tablelands)

The distribution of registrars and supervisors was primarily assessed using the Index of Access, previously developed nationally using 2012 GP supply data (GP’s Medicare billing) [18, 19]. It is an aggregate measure of spatial accessibility to GPs across rural Australia, calculated for small areas. In brief, it integrates multiple dimensions of access by simultaneously adjusting for geographical proximity, potential population demands, service availability, health needs and travel behaviours of populations. While considered a fit-for-purpose tool for measuring access to primary care, the Index of Access only been applied to workforce planning in one hypothetical case study [20]. A similar but modified approach, however, has been used in a national government report of access for Indigenous populations [21]. For ease of assessment in this study, the Index of Access scores (formatted as Provider-to-Population Ratios, PPRs) are collapsed into four ordinal groupings, namely level 1 (highest supply): > = 1:1250; level 2: < 1:1250 and > =1:1500; level 3: < 1:1500 and > =1:2000; and level 4 (lowest supply): < 1:2000.

An additional six publically available national indicators were selected to provide more information about supply and distribution relative to community need. Firstly, the District of Workforce Shortage (DWS) is an alternative binary measure of GP supply updated annually. For this paper, DWS status was aggregated for 10 years (2007–2017, missing in 2014) and measured in 2017 alone. The other five indicators were: Department of Health’s Modified Monash Model (MMM) rurality classification and four measures from the Australian Bureau of Statistics, namely the 2011 census population size data; ASGC-Remoteness Area (ASGC-RA); Socio-Economic Indicator For Areas (SEIFA); and the 2011 census proportion of the population that were Indigenous based. At the sub-regional level, an assessment against the expected level of registrars was made by determining whether their observed proportional distribution was within 2% of GP supply (2012 GP FTE) or population need (2011 census).

All indicators are coded so that ‘greatest need’ has the highest category. The DWS measure used quarter 1 status for each calendar year, and was coded 4 to 1 as ‘consistently DWS’ (9–10 of 10 years being undersupply / shortage), ‘majority DWS’ (6–8 of 10), ‘somewhat DWS’ (3–5 of 10) and ‘mostly not DWS’ (0–2 of 10 years). SEIFA (normally distributed with a mean of 1000 and standard deviation of 100) was coded using 4 levels: < 900, 900–950, 950–1000 and > 1000 while Indigenous population proportion (national average is 3.3%) was also coded using 4 levels: < 5%, 5–10%, 10–15 and > 15%. MMM (7 levels) and ASGC-RA (5 levels) used the original classification codes.

For regional-level assessment, supply *was assessed against expected levels by determining whether registrar or supervisor proportion is above 2%, within 2% (equal) or below 2% of either GP supply (2012 GP FTE) or demand (ABS 2011 population census).* For town-level assessment, each town was sorted according to its category code in some or all of the following indicators: (1) Index of Access – 4 levels; (2) DWS 2017 status; (3) DWS consistency 2007–17; (4) Rurality / remoteness; (5) Number of supervisors – ascending from 0; (6) Number of registrars – ascending from 0; (7) Population size – descending; (8) Proportion Indigenous; (9) Proximity to nearest town of > 5000 population.

Critically, the approach and outcomes were considered by expert panels with strong local health systems knowledge. Firstly, town- and region-level results were presented and discussed at project expert advisory group meetings, including the researchers and representatives from rural health workforce agencies, rural GPs, JCU and Hospital and Health Services. This process verified both our approach and reliability of our findings, as well as assisting interpretation of emerging patterns by putting the indicators into a practice context. Secondly, face to face meetings were hosted by the RTO executive team together with local GMT staff, health services and active supervisors, registrars and practice managers to discuss how the sub-region and town-level results impact on future strategic decisions about training posts.

## Results

**Fig. 1:**
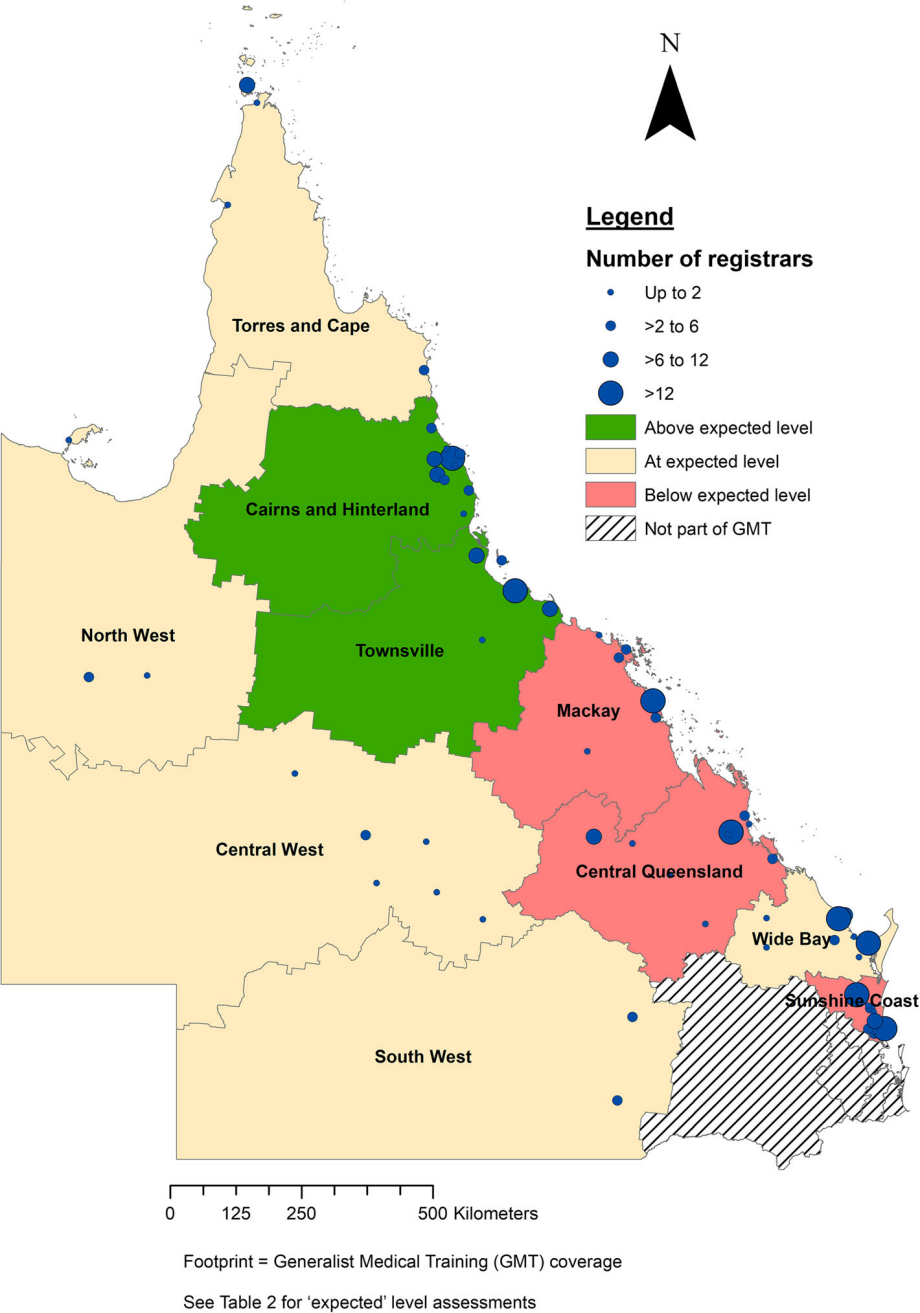
Distribution of GP registrars relative to population size in North West Queensland’s Hospital and Health Service regions

In 2017, 378 new and continuing registrars were participating in GP vocational training across 75 different towns. There were 582 supervisors with either primary or secondary supervisory roles. Most registrars were located in coastal areas, in Queensland’s larger regional centres (Fig. 1). Around 40 additional towns had active supervisors but only intermittent (visiting) registrars and thus are not identified as current registrar sites. A further 62 towns with a population greater than 500 (including 16 of population1000–3000) could not host registrars as their main site because there were no known GP services (measured in 2012), though further analysis found that 85% of these were located within 30 min driving time from a larger rural town (> 5000 population).

While about half of the sub-regions had the expected distribution of registrars given the regional population or GP supply, two sub-regions (Cairns and Hinterland/Tablelands) and Townsville) were relatively oversupplied with registrars and two sub-regions (Mackay, Central Queensland) relatively undersupplied. Sunshine Coast was also assessed as undersupplied for registrars; however it is also seen that same region has GP oversupply when compared to the population level. As shown in Table 2, Central Queensland contains 14% of the population of the whole region and 13% of services as measured by GP access but only 11% of registrars and 10% of supervisors are in this sub-region (‘Below expected level’). In contrast, Townsville sub-region has 15% of the population and 16% of services but it has 20% of both registrars and supervisors (‘Above expected level’).

**Table 2:** Distribution of registrars and supervisors compared to GP supply and population, by sub-region^a^

Sub-region	GP FTE in 2012 (Supply)*	ABS 2011 census population (Demand)	Registrars	Active Supervisors	Expected level assessment^b^
Cairns	13%	13%	15%	16%	Above
Central Qld	13%	14%	11%	10%	Below
Central West	1%	1%	3%	1%	Equal
Mackay	9%	11%	8%	8%	Below
North West	1%	2%	2%	3%	Equal
South West	2%	2%	2%	2%	Equal
Sunshine Coast	27%	23%	16%	18%	Below
Tablelands	3%	3%	6%	5%	Above
Torres & Cape	1%	2%	3%	2%	Equal
Townsville	16%	15%	20%	20%	Above
Wide Bay	15%	14%	15%	14%	Equal

*GP FTE (2012) = aggregation of GP supply (full-time equivalence) based on the Index of Access 2012 data; ABS = Australian Bureau of Statistics 2011 census

^a^Each column aggregates to 100% (i.e. the total study footprint)

^b^Expected level assessment: Whether registrar or supervisor % is above/within(equal)/below 2% of GP supply (GP FTE) or demand (ABS population census)

The four sub-regions with the lowest population density, namely South West, Central West, North West and Torres and Cape, were each assessed as being at expected levels of registrars. Table 2 reveals that the proportion of registrars in each of these regions was 0–2% above the population and Index proportions, suggesting that distribution of registrars into these priority regions is satisfactory.

Table 3 shows the relative distribution of the population compared with distribution of registrars for the six key indicators. Our primary indicator, the Index of Access, revealed a good distribution of registrars into the most problematic access areas (Level 4). Almost a quarter of registrars (24%) were training in areas with the lowest relative access to primary care, compared with 15% of the population being in these areas. In contrast, registrars are notably less likely to be working in the Level 1 ‘access’ towns (mostly located in the metropolitan Sunshine Coast region). Similarly, proportionally more registrars were working in locations that were either consistently DWS (23% of registrars) or mostly DWS (10% of registrars) than would be expected if registrars were distributed in line with the population in these areas (total 23%).

**Table 3:** Distribution of the population and registrars in the study’s geographical footprint against key indicators

Indicator	← Most rural / ‘greatest need’ indicator			Least rural / ‘less need’ indicator →			
Index of Access category	Level 4 (< 1:2000)	Level 3 (< 1:1500 & > 1:2000)	Level 2 (< 1:1250 & > 1:1500)	Level 1 (> 1:1250)			
Proportion of the population	15%	26%	38%	21%			
Proportion of GP registrars	24%	26%	40%	10%			
MMM category	MMM-7	MMM-6	MMM-5	MMM-4	MMM-3	MMM-2	MMM-1
Proportion of the population	3%	3%	9%	8%	10%	49%	18%
Proportion of GP registrars	7%	3%	12%	15%	7%	49%	7%
ASGC-RA	RA-5 (very remote)	RA-4 (remote)	RA-3 (outer regional)	RA-2 (inner regional)	RA-1 (major cities)		
Proportion of the population	2%	3%	38%	40%	16%		
Proportion of GP registrars	6%	4%	47%	38%	5%		
DWS (2007–17)	4: Consistently DWS	3: Mostly are DWS	2: Somewhat DWS	1: Mostly not DWS		2: 2017 = DWS	1: 2017 = not DWS
Proportion of the population	19%	4%	16%	61%		35%	65%
Proportion of GP registrars	23%	10%	12%	54%		40%	60%
Proportion of population who are Indigenous	4: > 15%	3: 10–15%	2: 5–10%	1: < 5%			
Proportion of the population	5%	4%	35%	56%			
Proportion of GP registrars	12%	5%	41%	42%			
SEIFA	4: < 900	3: 900–950	2: 950–1000	1: > 1000			
Proportion of the population	8%	23%	27%	42%			
Proportion of GP registrars	9%	34%	28%	28%			

*ASGC-RA* Australian Standard Geographical Classification – Remoteness Areas, *DWS* District of Workforce Shortage, *MMM* Modified Monash Model, *SEIFA* Socioeconomic Indicator For Areas

Overall, MMM-4, MMM-5 and MMM-7 locations each had more registrars than expected. In contrast, MMM-3 and MMM-1 (metropolitan) had below expected numbers of registrars. Similar outcomes are revealed by the ASGC-RA classification, with outer regional, remote and very remote locations having more registrars than expected compared with the population distribution. Communities with a high Indigenous proportion (> 15%) had more than double the expected supply of registrars in their communities, while communities with lower SEIFA scores than average (< 1000) also had more registrars than expected based on population distribution.

Table 4 shows an anonymised listing of town-level indicators, for about 10% of randomly-selected towns (population > 500) in the whole region. The identifiable version of this table (not shown), containing all current and potential registrar and supervisor locations, was used to determine which specific towns should be prioritised for future registrar and supervisor development. To illustrate, communities with both poor access (Index of Access score 4) and long-term designated workforce undersupply (DWS 2007–2017 score 4) were initially identified (towns 1 to 8, Table 4). To further reduce the number of prioritised towns, an additional indicator for the proportion of the population that is Indigenous was introduced, narrowing the most highly prioritised communities to towns 2, 4 and 7.

**Table 4:** Individual town-level assessment of key indicators

Town(s)	2011 population	Sub-region	ASGC-RA	MMM	Registrar count	DWS 2007–17	Index of Access	SEIFA	Indigenous
Town 1	1501–5000	Mackay	3	5	< 2	4	4	1	2
Town 2	< 1500	Cape & Torres	5	6	< 2	4	4	4	4
Town 3	< 1500	South West	4	7	< 2	4	4	3	3
Town 4	< 1500	Cape & Torres	5	7	< 2	4	4	4	4
Town 5	< 1500	Central West	4	7	< 2	4	4	2	2
Town 6	< 1500	Central West	5	7	2–5	4	4	3	4
Town 7	< 1500	North West	5	7	< 2	4	4	4	4
Town 8	5001–15,000	Townsville	3	4	2–5	4	4	3	4
Town 9	1501–5000	Cairns	3	5	2–5	2	4	4	4
Town 10	< 1500	Wide Bay	2	5	< 2	1	4	2	1
Town 11	5001–15,000	Tablelands	3	4	6–10	1	4	3	3
Town 12	1501–5000	Cape & Torres	5	6	2–5	4	3	3	4
Town 13	5001–15,000	Central Qld	2	2	< 2	2	3	1	3
Town 14	5001–15,000	Townsville	3	4	6–10	4	2	3	3
Town 15	1501–5000	Sunshine Coast	2	2	< 2	2	1	1	1

*ASGC-RA* Australian Standard Geographical Classification – Remoteness Areas, *DWS* District of Workforce Shortage, *MMM* Modified Monash Model, *SEIFA* Socioeconomic Indicator For Areas

## Discussion

Our study demonstrates a potentially useful health workforce planning method for informing improved GP distribution to promote more equitable access to healthcare. Multiple publicly available national-scale indicators were used to assess the distribution of registrars and supervisors within the geographical footprint of a single RTO. Most of these methods were simple proportions based on the most updated source of data. An additional two indicators used were: the Index of Access, and an aggregated score of DWS status over a 10 year period. However, the indicator measuring the proportion of the Indigenous population proved useful in identifying several of the highest priority communities for targeting expansion of registrar and supervision posts. The remaining indicators complemented the information provided by these key indicators, together providing a suite of evidence to inform workforce planning by helping to discern the factors related to inequitable access.

These results demonstrate that overall distribution was already good in GMT’s area, with the ‘most rural’ and ‘greatest need’ locations having more registrars than expected, whilst also seeing fewer than expected registrars training in metropolitan areas in 2017. However, through discussion with the expert panel, they also provided a finer level of knowledge for achieving improved distribution, objectively identifying priority towns for future targeting and service planning. Improving the distribution of rural health training relative to long-term workforce and population need is critical for developing a workforce with the right skills, addressing equity of access to healthcare and improving population health outcomes. Successfully achieving these outcomes relies heavily on organisations committed to the appropriate distribution and grounded medical workforce planning processes that are best achieved by collaborations between workforce researchers and training providers. The results had a practical application by informing training planning decisions of where to build registrar supervision. There is very limited evidence of the use of similar health service planning methods in the published, peer-reviewed literature [22, 23], although it is possible that other approaches are used but not published.

The utility of the quantitative analysis was most powerful as the starting point for conversations with local experts. The people who are managing and coordinating training at the coalface have a strong understanding of the local context and this supported the interpretation of the findings. The indicators provided the basis for an informed discussion about why specific communities may or may not have service levels above, at, or below expected levels and the main drivers thought to explain the current distribution. A range of explanations were identified by local experts including relating to history, geography, local relationships, individuals, private investment, population demographics, access in neighbouring towns and specific community health needs. This additional contextual knowledge clarified where unexpected poorer supply was observed and is critical for determining whether additional resources or efforts by the RTO are likely to achieve their goals. For example, the quantitative analysis identified that a number of towns had lower than expected registrars, but targeting more training posts at these locations was not considered a priority as other indicators like DWS, SEIFA and Indigenous were not similarly highly rated. In contrast, many towns, often in more remote areas, had registrars at expected levels but other indicators such as Indigenous proportions, SEIFA and isolation from larger rural towns suggested that their local health needs would be well above average. However, the smaller nearby towns served by these larger rural towns were frequently identified as having poorer access, suggesting that the medical workforce was generally undersupplied across these remote areas.

Our study was conducted in partnership with an RTO explicitly encouraging more registrars to work in underserved areas and directly linked to a university with a socially accountable medical school, thus it was not surprising that sub-regional distributional results were largely encouraging. However, the collaborative approach to planning between workforce academics and program managers has strengthened this study’s ability to translate findings to the development of material specifically targeting newly identified underserved areas and a more prioritised approach to registrar recruitment and placements. It is expected that this full planning cycle would only need to be repeated every 3–4 years to adequately inform strategic directions, check in on community changes and monitor progress towards achieving the social accountability goals of the GP training program. By project conclusion, key identified strategies were identified by the research team and expert panels for future implementation:

1. Identify activities and opportunities to improve the pipeway for medical students and junior doctors to remote GP training experiences
2. Develop strategies to attract and retain registrars and supervisors to GP training in identified underserved communities
3. Establish opportunities for improved support and self-care for registrars and supervisors in remote communities

It is important to acknowledge the distinction between local workforce requirements being met by registrars in a ‘service learning’ capacity (i.e. short-term workforce supply provided by AGPT), and the more long-term contribution to sustainable workforce achieved by exposing registrars to underserviced towns, and ensuring adequate infrastructure and support (including supervisor access) which provides an incentive for registrars to remain in or return to those towns after fellowship training. Local experts within the RTO could use the evidence-based approach outlined in this paper to not only build incentives for registrar placement in underserviced towns, but also work with local communities, health services and government to address sustainable provision of general practice workforce in these towns.

Key limitations of this study are that the Index of Access is based on data that are now somewhat outdated (2012). Its method complexity and limited availability of updated data prevented an update being feasible for this study, though spatial patterns are unlikely to change significantly across different periods [18]. In this study 2011 census population data were used as the alternative measure against which to assess distribution as 2016 census data were not yet available when this study commenced. Using population data may not accurately reflect population health care needs as factors such as age profile, gender and Indigeneity, which impact on health, are not accounted for. We counteracted these concerns by using multiple indicators which provide a nuanced lens of outputs that conferred highly with GP supply results. The process of assessing these indicators against registrar and supervisor data was non-trivial, supported by a research grant, and likely not practical to conduct on a regular routine basis.

## Conclusions

This paper demonstrates a new planning approach for ensuring training posts address community need. We show the utility of a suite of indicators to inform and monitor distributed GP training, an approach which is widely applicable elsewhere. Whilst these results confirmed that registrar training distribution to address community need is already strong in this region, finer examination of different indicators identified avenues for further improvement. This study’s quantitative analysis suite was most powerful for enabling conversations with local experts and the approach was strengthened by drawing on their interpretation of the data in the local context and assisting to plan solutions. The demonstrated approach is considered highly applicable for other similar entities, enabling distributed workforce planning and monitoring in a range of contexts, with increased sensitivity for prioritising distributed training for long-term workforce goals of equitable healthcare, and for supporting useful discussions about the potential causes and solutions.

## Abbreviations

ABS: Australian Bureau of Statistics
AGPT: Australian General Practice Training
ASGC-RA: Australian Standard Geographical Classification – Remoteness Areas
DWS: District of Workforce Shortage
GMT: Generalist Medical Training
GP: General practitioner
JCU: James Cook University
MMM: Modified Monash Model
RTO: Regional Training Organisation
SEIFA: Socio-Economic Indicator For Areas

## References

[1] Dussault G, Franceschini MC (2006). Not enough there, too many here: understanding geographical imbalances in the distribution of the health workforce. Hum Resour Health.

[2] Australian Government Department of Health and Ageing. National Strategic Framework for Rural and Remote Health. 2011. Available from: http://www.health.gov.au/internet/main/publishing.nsf/Content/national-strategic-framework-rural-remote-health.

[3] Mason J (2013). Review of Australian government health workforce programs.

[4] Australian Bureau of Statistics (2017). Health service usage and health related actions, Australia 2014–15.

[5] Australian Institute of Health and Welfare (2017). Rural and remote health: Web report.

[6] Australian Government Department of Health (2018). General practitioner workforce statistics 2016–17.

[7] Strasser R, Neusy A-J (2010). Context counts: training health workers in and for rural and remote areas. Bull World Health Organ.

[8] Wilson N, Couper I, De Vries E, Reid S, Fish T, Marais B (2009). A critical review of interventions to redress the inequitable distribution of healthcare professionals to rural and remote areas. Rural Remote Health.

[9] Australian Government Department of Health (2018). Australian general practice training handbook.

[10] Murray RB, Larkins S, Russell H, Ewen S, Prideaux D (2012). Medical schools as agents of change: socially accountable medical education. Med J Aust.

[11] Sen Gupta T, Wolley T, Murray R, Hays RB, Staer T (2014). Positive impacts on rural and regional workforce from the first seven cohorts of James Cook University medical graduates. Rural Remote Health.

[12] Bailey BES, Wharton RG, Holman CDAJ (2016). Glass half full: survival analysis of new rural doctor retention in Western Australia. Aust J Rural Health.

[13] Wilkinson D, Laven G, Pratt N, Beilby J (2003). Impact of undergraduate and postgraduate rural training, and medical school entry criteria on rural practice among Australian general practitioners: national study of 2414 doctors. Med Educ.

[14] Campbell DG, Greacen JH, Giddings PH, Skinner LP (2011). Regionalisation of general practice training — are we meeting the needs of rural Australia?. Med J Aust.

[15] McGrail MR, Russell D, Campbell D (2016). Vocational training of general practitioners in rural locations is critical for Australian rural medical workforce supply. Med J Aust.

[16] Rees GH, Crampton P, Gauld R, MacDonnell S (2018). The promise of complementarity: using the methods of foresight for health workforce planning. Health Serv Manag Res.

[17] Lopes MA, Almeida AS, Almada-Lobo B (2015). Handling healthcare workforce planning with care: where do we stand?. Hum Resour Health.

[18] McGrail MR, Humphreys JS (2015). Spatial access disparities to primary health care in rural and remote Australia. Geospat Health.

[19] McGrail MR, Humphreys JS. Development of a national index of access for primary health care in Australia: discussion paper: Monash University; 2015.

[20] McGrail MR, Russell DJ, Humphreys JS (2017). Index of access: a new innovative and dynamic tool for rural health service and workforce planning. Aust Health Rev.

[21] Australian Institute of Health and Welfare (2014). Access to primary health care relative to need for indigenous Australians.

[22] Hagopian A, Mohanty MK, Das A, House PJ (2012). Applying WHO’s ‘workforce indicators of staffing need’ (WISN) method to calculate the health worker requirements for India's maternal and child health service guarantees in Orissa State. Health Policy Plann.

[23] Krause DD (2015). State health mapper: an interactive, web-based tool for physician workforce planning, recruitment, and health services research. Southern Med J.

